# Spatiotemporal Variability in Phosphorus Species in the Pearl River Estuary: Influence of the River Discharge

**DOI:** 10.1038/s41598-017-13924-w

**Published:** 2017-10-20

**Authors:** Ruihuan Li, Jie Xu, Xiangfu Li, Zhen Shi, Paul J. Harrison

**Affiliations:** 1 State Key Laboratory of Tropical Oceanography, South China Sea Institute of Oceanology, Chinese Academy of Sciences, 164 West Xingang road, Guangzhou, 510301 China; 2 College of Earth Sciences, University of Chinese Academy of Sciences, Beijing, 100049 China; 3 Department of Earth and Ocean Sciences, University of British Columbia, Vancouver, BC V6T 1Z4 Canada

## Abstract

Phosphorus was the stoichiometrically limiting nutrient in the Pearl River Estuary (PRE). In order to examine how the river discharge regulates phosphorus dynamics in the PRE, the concentrations of dissolved inorganic phosphorus (DIP) and organic phosphorus (DOP), particulate inorganic phosphorus (PIP) and organic phosphorus (POP) in the water column were determined in May 2015 (spring), August 2015 (summer) and January 2016 (winter). Our results showed that all types of phosphorus were significantly lower in spring and summer than in winter. The Pearl River discharge input played an important role in regulating phosphorus dynamics. Strong vertical mixing in winter resulted in high levels of total particulate phosphorus (1.50 ± 0.97 μM) and dissolved phosphate (DIP: 1.44 ± 0.57 μM, DOP: 0.58 ± 0.42 μM) at the surface. On the other hand, the river discharge input created stratification in spring and summer, favoring the settlement of suspended particulate matter and enhancing light levels. This promoted phytoplankton growth, which was responsible for a DIP drawdown of 0.43 ± 0.37 μM in May and 0.56 ± 0.42 μM in August at the surface. Additionally, stratification restricted the bottom phosphorus replenishment. Our findings provided an insight into processes causing stoichiometric P limitation in the PRE.

## Introduction

Phosphorus (P) is an important nutrient for all living organisms and plays an essential role in regulating the primary production in estuarine and marine environments^1^. Primary production is frequently limited by P in estuaries and marine systems^2–4^.

Phosphorus is present in both dissolved and particulate organic or inorganic forms in aquatic environments. The various P species differ in bioavailability and geochemical cycling in the water column. Dissolved inorganic P (DIP) is preferentially utilized by living organisms^5,6^. Dissolved organic P (DOP) represents an intermediate state during the mineralization of particulate organic matter and is a potential P source for plankton^7^. Marine organisms not only uptake inorganic phosphate but also utilize part of DOP under specific ecological conditions, especially when the supply of DIP is not sufficient^8–11^. For example, 55–65% of DOP was found to be bioavailable in the productive surface layer of the central Baltic Sea^12^, up to 88% in Loch Creran (Scotland)^13^, 7–25% in the North Pacific Subtropical Gyre^3^ and 8% in Bothnian Bay^9^.

It has been reported that more than 90% of phosphorus carried by rivers to estuaries and coastal waters is associated with suspended solids^14,15^. As a result, particle-bound phosphorus is expected to be an important fraction of phosphorus in estuaries. Particulate phosphorus (PP) consists of living and dead plankton, precipitates of P minerals, P adsorbed to particles, and amorphous P phases^16^. Riverine PP exists as particulate inorganic phosphorus (PIP) and particulate organic phosphorus (POP). POP originates from the living or detrital organic matter. However, the components of PIP are very complex. It encompasses DIP adsorbed onto particles and phosphorus co-precipitated with calcite or iron oxyhydroxides^6^. Approximately 20% of PP in estuaries is DIP adsorbed onto particles^17^, which is desorbed to water through biogeochemical processes. Furthermore, increasing salinity improves the desorption of DIP adsorbed onto particles^15^. Additionally, PP that is bound to oxidized iron species^18^ may release DIP to water when iron oxyhydroxide is reduced from suspended particulate matter (SPM) and sediments^15^, which enhances DIP availability in aquatic environments^19^. Hence, PP is also a potential source for the phytoplankton growth in estuaries. It is necessary to assess the relative contribution of phosphorus other than DIP to the phosphorus pool in aquatic environments, especially in P-limited waters.

**Figure 1:**
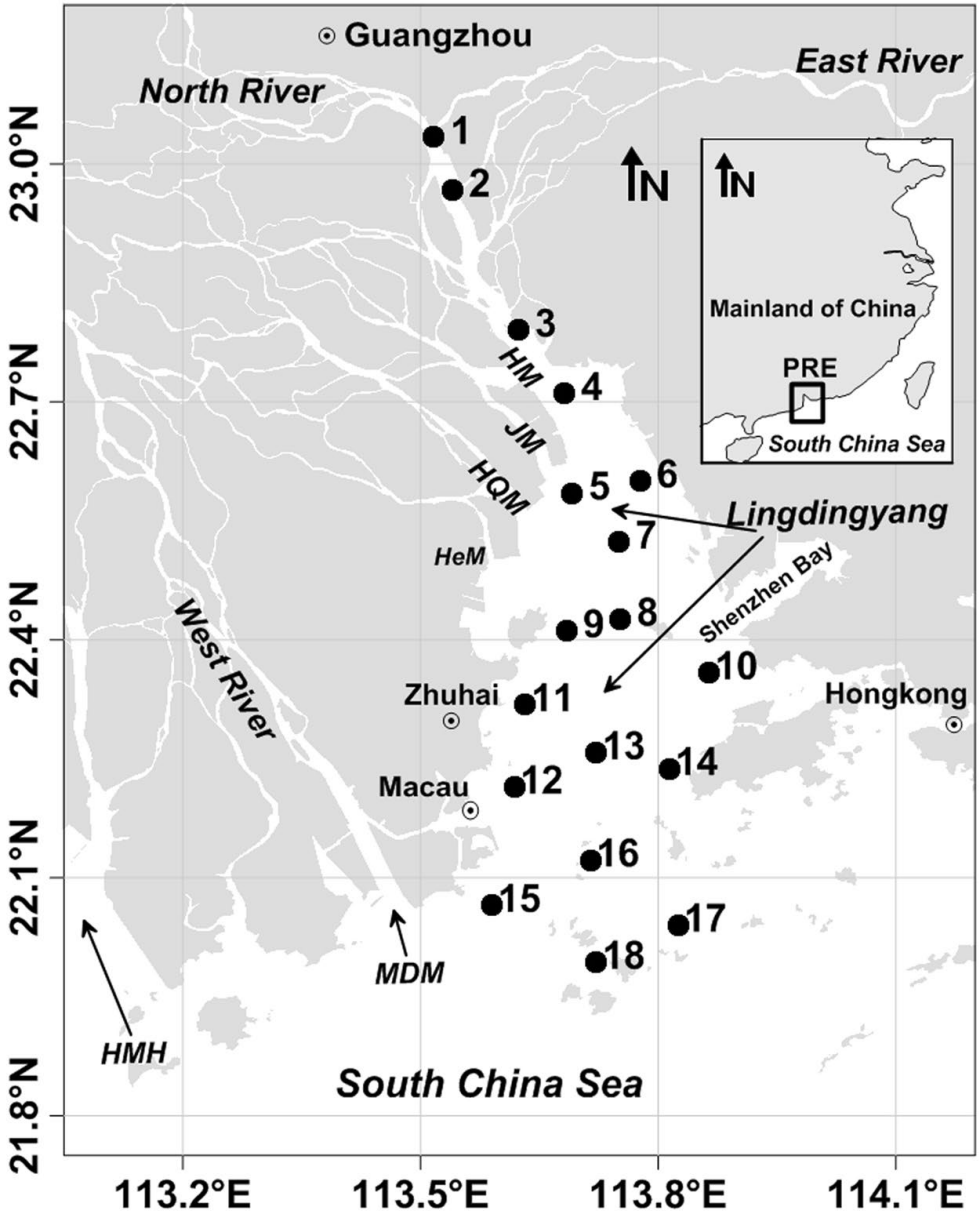
Location map of the Pearl River estuary and sampling stations for the three cruises during 2015–2016. The solid circles denote the sampling stations. PRE, HM, JM, HQM, HeM, MDM and HMH represent the Pearl River estuary, Humen, Jiaomen, Hongqimen, Hengmen, Maodaomen and Huangmaohai, respectively. The inset at the upper right is the location map, which was plotted using Surfer 11 (http://www.goldensoftware.com/products/surfer).

The Pearl River (PR), located along the northern boundary of the South China Sea (SCS) (Fig. 1), is the third longest river (2200 km) in China with a drainage area of 453,700 km^2^ ^20^. The regional climate is dominated by southwesterly/northeasterly monsoon winds in summer/winter, with an annual rainfall from 1600 to 2300 mm^21^. The annual river discharge from the PR is approximately 3.3 × 10^11^ m^3^ yr^−1^, ~80% of which is delivered during the wet season (April–September)^22^. The PR’s maximum discharge occurs in summer^23^, and it carries an annual sediment load of 85 × 10^6^ tons y^−1^ into the SCS^20^. It is estimated that half of the PR freshwater discharges into the Lingdingyang (LDY) through four northeastern outlets (Humen (HM), Jiaomen (JM), Hongqimen (HQM) and Hengmen (HeM))^20^ (Fig. 1).

The PR delta is the fastest-developing region in southern China. Large amounts of wastewater and pollutants are discharged into the PRE without proper treatment due to the rapid development of the economy^2^. Meanwhile, large amounts of nutrients are transported to the coastal region through the river. Previous studies have shown that nitrate concentrations in the PR are extremely high (up to 100 μM), while phosphate concentrations are relatively low (~1 μM)^23^, resulting in potential P limitation in the estuarine and coastal plume^24^. Much attention has been paid to the nutrient levels, nutrient fluxes at the sediment-water interface, physical processes and harmful algal blooms in the estuary^25–27^. However, little is known about the dynamics of various P species in the PRE. The present study investigated the concentrations of various P species in the water column in three seasons, in order to examine how the Pearl River discharge regulates the dynamics of various P species in the PRE.

## Results

### Hydrographic properties

**Figure 2:**
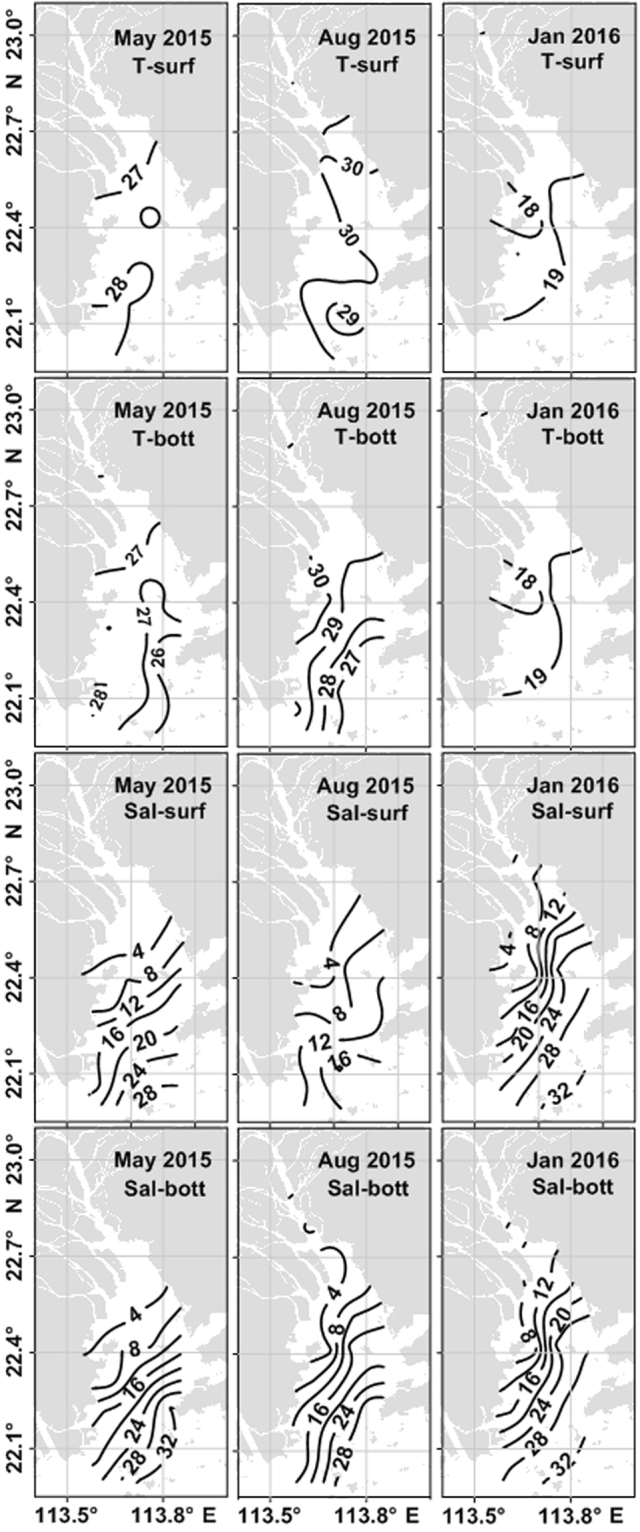
Horizontal distributions of temperature (°C) and salinity (psu) at the surface and the bottom in the PRE in May 2015, August 2015 and January 2016. T, Sal, surf, bott, Jan and Aug, represent temperature, salinity, surface, bottom, August and January, respectively. The figure was plotted using Surfer 11 (http://www.goldensoftware.com/products/surfer).

The surface water temperature increased from the upper to lower estuary in May 2015 and January 2016 but decreased in August 2015 (Fig. 2). In May 2015, the surface temperature was similar to that in the bottom layer in the HM channel (between Station 1 and Station 6) but was 0.3 to 1.4 °C higher in the middle and lower estuary (between Station 7 and Station 18). The seasonal thermocline formed in May and was enhanced in August (Δ*t* ranged from 0.2 to 4.7 °C between Station 1 and Station 18) (Fig. 2). Salinity at the surface and bottom gradually increased from the upper to the lower estuary (Fig. 2). Salinity at the surface exhibited clear seasonal variability, with a minimum (<16 psu) in August 2015 and a maximum (up to 32 psu) in January 2016. The largest difference (~12 psu) in salinity between surface and bottom occurred in August 2015. The water column was mixed well in January, while stratification occurred in May and August 2015.

The SPM concentrations were the highest (25.5 mg L^−1^ at the surface and 49.4 mg L^−1^ at the bottom) in winter (January 2016), moderate in spring (May 2015) and the lowest (10.5 mg L^−1^ at the surface and 14.6 mg L^−1^ at the bottom) in summer (August 2015).

### Temporal variations of dissolved inorganic nitrogen and silicate

**Figure 3:**
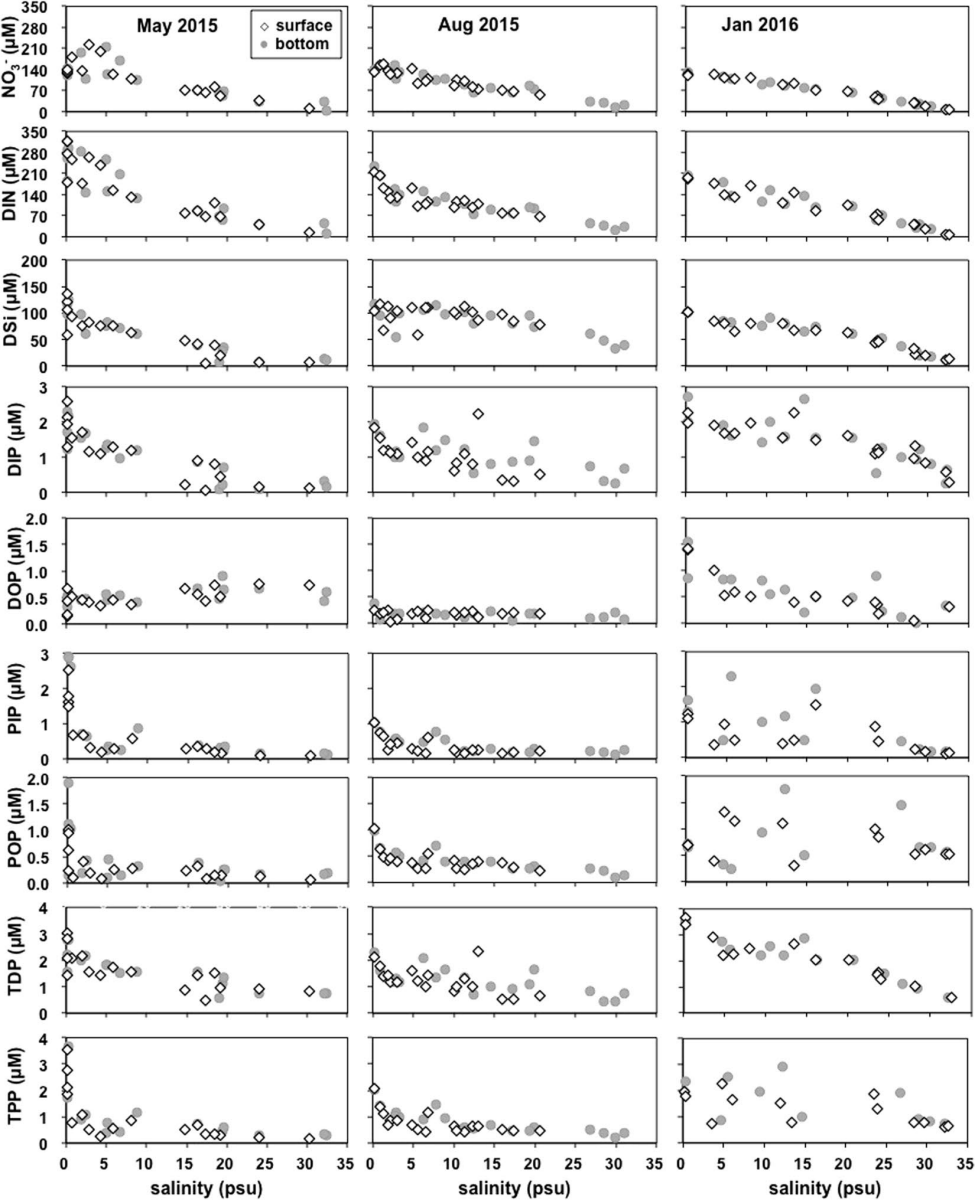
Variations in the concentrations of NO_3_^−^, DIN, DSi, DIP, DOP, PIP, POP, TDP and TPP with salinity in the PRE at the surface and the bottom in May 2015, August 2015 and January 2016.

Temporal variability in nitrate (NO_3_^−^), dissolved inorganic nitrogen (DIN, the sum of NO_3_^−^, nitrite (NO_2_^−^) and ammonium (NH_4_^+^)) and silicate (DSi) concentrations was obvious in the PRE (Fig. 3). The concentrations of NO_3_^−^ and DSi declined from the upper to lower estuary (Fig. 3). NO_3_^−^ was the dominant species of DIN, accounting for 58–97% of DIN in the water column, except in May 2015 (39–93% of DIN). DIN had a significant relationship with salinity, especially in January 2016 (p < 0.01, Table 2). Similarly, a significant correlation between salinity and DSi was observed (Table 2, p < 0.01) (Fig. 3).

**Table 2:** The relationship between DIN and DSi vs. salinity, PIP vs. SPM and POP vs. Chl *a* at the surface water.

Time	Relational expression	R^2^
Spring (May 2015)	y = −5.0661x + 160.31 (DIN vs. salinity)	0.73
	y = −3.6168x + 97.04 (DSi vs. salinity)	0.79
	y = 0.0265x + 0.0808 (PIP vs. SPM)	0.56
Summer (Aug 2015)	y = −5.6033x + 169.7 (DIN vs. salinity)	0.71
	y = −0.6469x + 102.54 (DSi vs. salinity)	0.05
	y = 0.021x + 0.0841 (PIP vs. SPM)	0.87
	y = 0.0103x + 0.2624 (POP vs. SPM)	0.55
	y = 0.0251x + 0.2322 (POP vs. Chl *a*)	0.75
Winter (Jan 2016)	y = −15.63x + 445.52 (DIN vs. salinity)	0.88
	y = −4.1402x + 126.47 (DSi vs. salinity)	0.87
	y = 0.0138x + 0.2601 (PIP vs. SPM)	0.66

### Temporal and spatial variations of DIP, DOP and nutrient ratios

DIP concentrations decreased with increasing salinity in the three cruises. There was little vertical variation in DIP and DOP. The highest DIP and DOP were found in January (DIP: ~1.43 µM, DOP: ~0.49 µM), followed by May and August (DIP: ~1.07 µM, DOP: ~0.18 µM).

**Figure 4:**
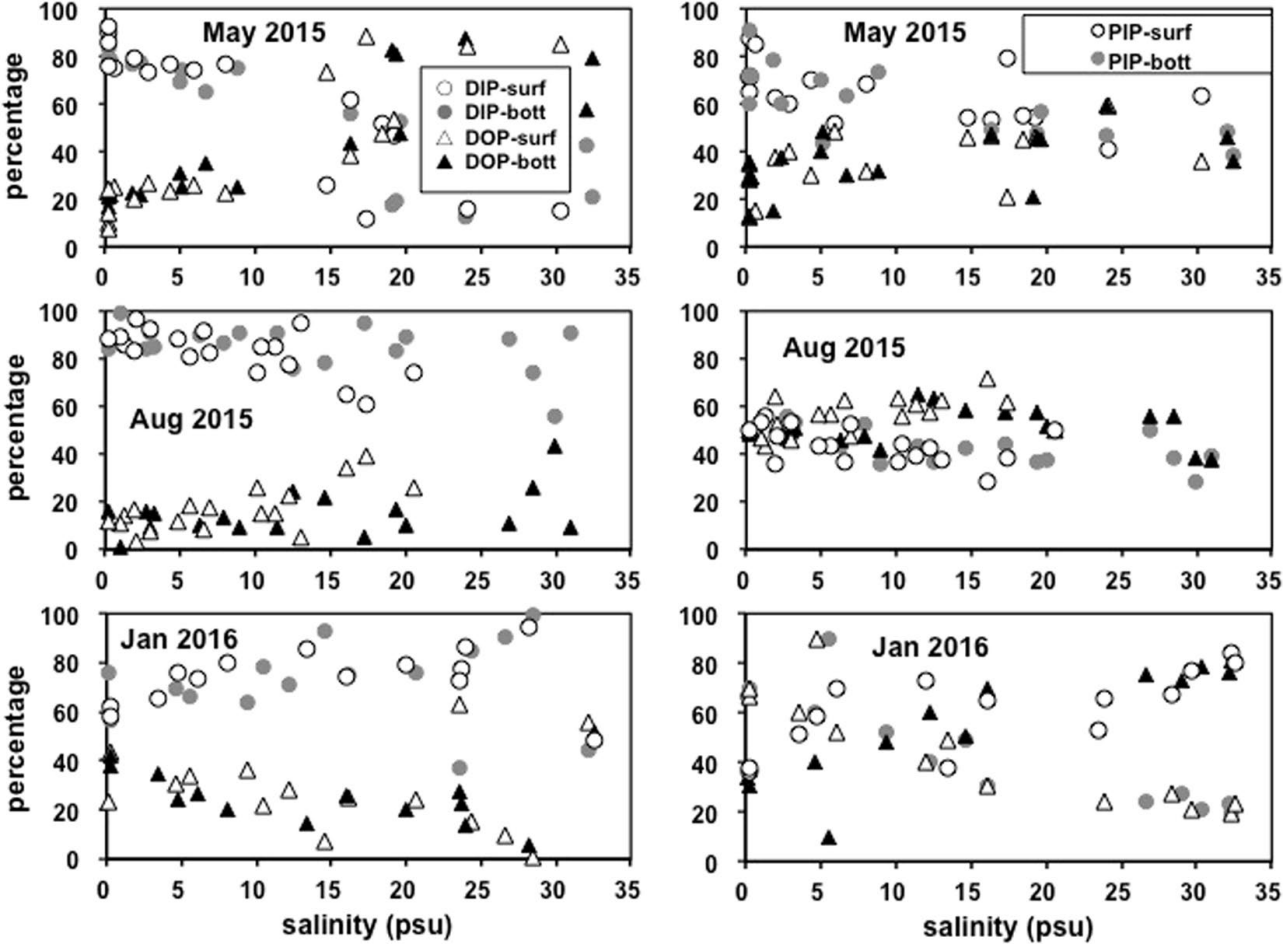
Variations in the relative contribution of DIP to the TDP pool and the relative contribution of PIP to the TPP pool with salinity in the PRE from the surface and bottom samples during investigations.

The relative contribution of DIP to TDP differed among the three cruises. In May, there was a shift from the dominance of DIP in low-salinity waters (S < 15 psu) to the dominance of DOP in high-salinity waters (S > 15 psu). In August, DIP dominated TDP, and the relative contribution of DIP to TDP remained relatively constant throughout the estuary. In January, DIP was the dominant species, and the ratio of DIP to TDP slightly decreased with increasing salinity (Fig. 4).

**Figure 5:**
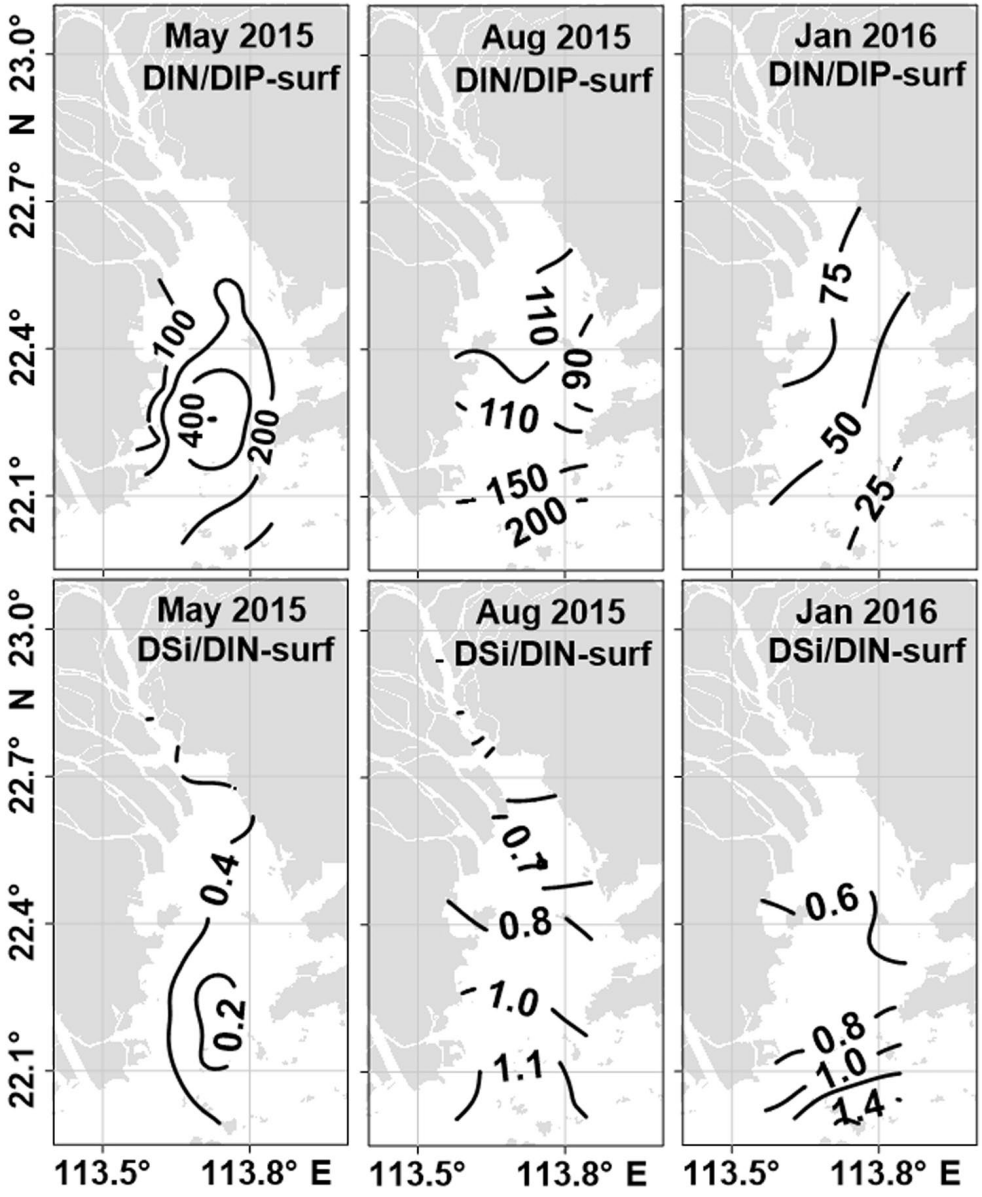
Horizontal distribution of nutrient ratios at the surface in the PRE in May 2015, August 2015 and January 2016. The map in this figure was plotted using Surfer 11 (http://www.goldensoftware.com/products/surfer).

The DIN:DIP ratios at the surface in the estuary varied between 15:1 and 330:1 mol mol^−1^ (except for 1163:1 mol mol^−1^ at Station 13 in May 2015), with an average of 101 ± 61:1 mol mol^−1^ (Fig. 5). The molar ratios of Si:DIN ranged from 0.16:1 mol mol^−1^ to 3.25:1 mol mol^−1^, with an average of 0.66 ± 0.51:1 mol mol^−1^ (Fig. 5), and increased from the upper to lower estuary, except for May 2015.

### Variability of PP

The mean total PP (TPP, as the sum of PIP and POP) concentrations were the highest (1.50 ± 0.97 μM at the surface and 1.93 ± 0.97 μM at the bottom) in January 2016, followed by those in May 2015 (0.99 ± 0.98 μM at the surface and 1.30 ± 1.43 μM at the bottom), then August 2015 (0.78 ± 0.42 μM at the surface and 0.83 ± 0.47 μM at the bottom). The TPP generally decreased from the upper to the lower estuary (Fig. 3). The ratio of TPP to SPM was significantly lower in May 2015 (mean of 1.48 ± 0.98 mg P g^−1^ at the surface and 1.27 ± 0.57 mg P g^−1^ at the bottom) than in the other periods.

The average PIP concentrations reached a maximum (0.68 ± 0.24 μM at the surface and 0.87 ± 0.26 μM at the bottom) in May 2015 and a minimum (0.36 ± 0.24 μM at the surface and 0.41 ± 0.26 μM at the bottom) in August 2015. The PIP concentrations were significantly correlated with SPM (Table 2, p < 0.01), both at the surface and the bottom, but not with chlorophyll *a* (Chl *a*) (p > 0.05). However, the POP concentrations in May (average of 0.31 ± 0.29 μM at the surface and 0.43 ± 0.49 μM at the bottom) were the lowest during the study period. POP had a significant relationship with Chl *a* and SPM both at the surface and the bottom in August 2015 (Table 2, p < 0.01).

TPP was dominated by PIP in May 2015 (Fig. 4), which contributed 64 ± 12% and 62 ± 16% to TPP at the surface and the bottom, respectively. In contrast, during August 2015 and January 2016, POP dominated TPP. In August 2015, POP made up 58 ± 6% and 53 ± 9% of TPP at the surface and the bottom in the regions beyond the upper estuary (S > 5 psu), respectively. PIP accounted for 39 ± 16% of TPP at the surface and 44 ± 22% at the bottom in January 2016.

### Dynamics of chlorophyll *a*

**Figure 6:**
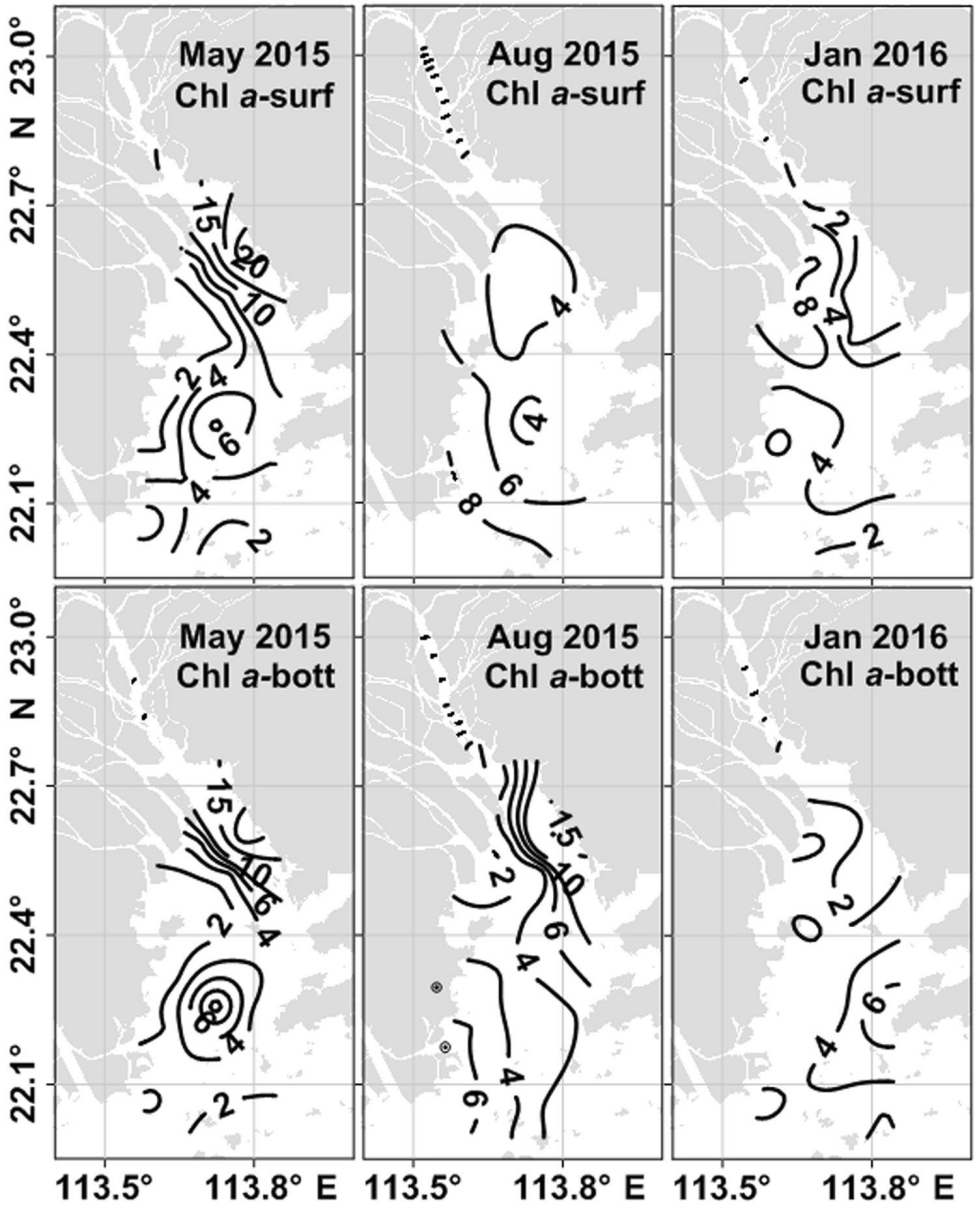
Horizontal distribution of chlorophyll *a* concentration (Chl *a*, μg L^−1^) at the surface and the bottom in May 2015, August 2015 and January 2016. The map in this figure was plotted using Microsoft office 2013 (https://products.office.com/).

Chl *a* concentrations varied considerably among different cruises (Table 1), and the horizontal distribution of Chl *a* was characterized by patchiness (Fig. 6). In May 2015, Chl *a* was, on average, 5.5 μg L^−1^, and a peak occurred at Station 6 (~29 μg L^−1^) (Fig. 6). The observed Chl *a* concentrations were higher in August 2015 both at the surface and near the bottom than in the other cruises (Table 1), and a maximum (~29 μg L^−1^) occurred at Station 1. In January 2016, the concentration of Chl *a* (average of 4.2 ± 3.3 μg L^−1^ at the surface and 3.7 ± 2.6 μg L^−1^ at the bottom) was the lowest during this study period.

**Table 1:** Temporal variations in suspended particulate matter (SPM) and chlorophyll *a* (Chl *a*) concentrations in the Pearl River estuary during the study period.

Season	SPM (mg L^−1^)		Chl *a* (μg L^−1^)	
	surface	bottom	surface	bottom
Spring (May 2015)	2.87–74.6 (22.6)	5.85–75.4 (31.1)	0.7–29 (6.8)	0.8–23 (5.8)
Summer (Aug 2015)	3.60–21.4 (10.5)	4.65–38.6 (14.6)	2.9–29 (7.4)	1.7–30 (7.5)
Winter (Jan 2016)	5.49–103 (25.5)	5.84–154 (49.4)	0.9–11.4 (4.2)	0.9–9.1 (3.7)

Mean values are given in parentheses.

### Variations in partitioning coefficient (K_d_)

**Figure 7:**
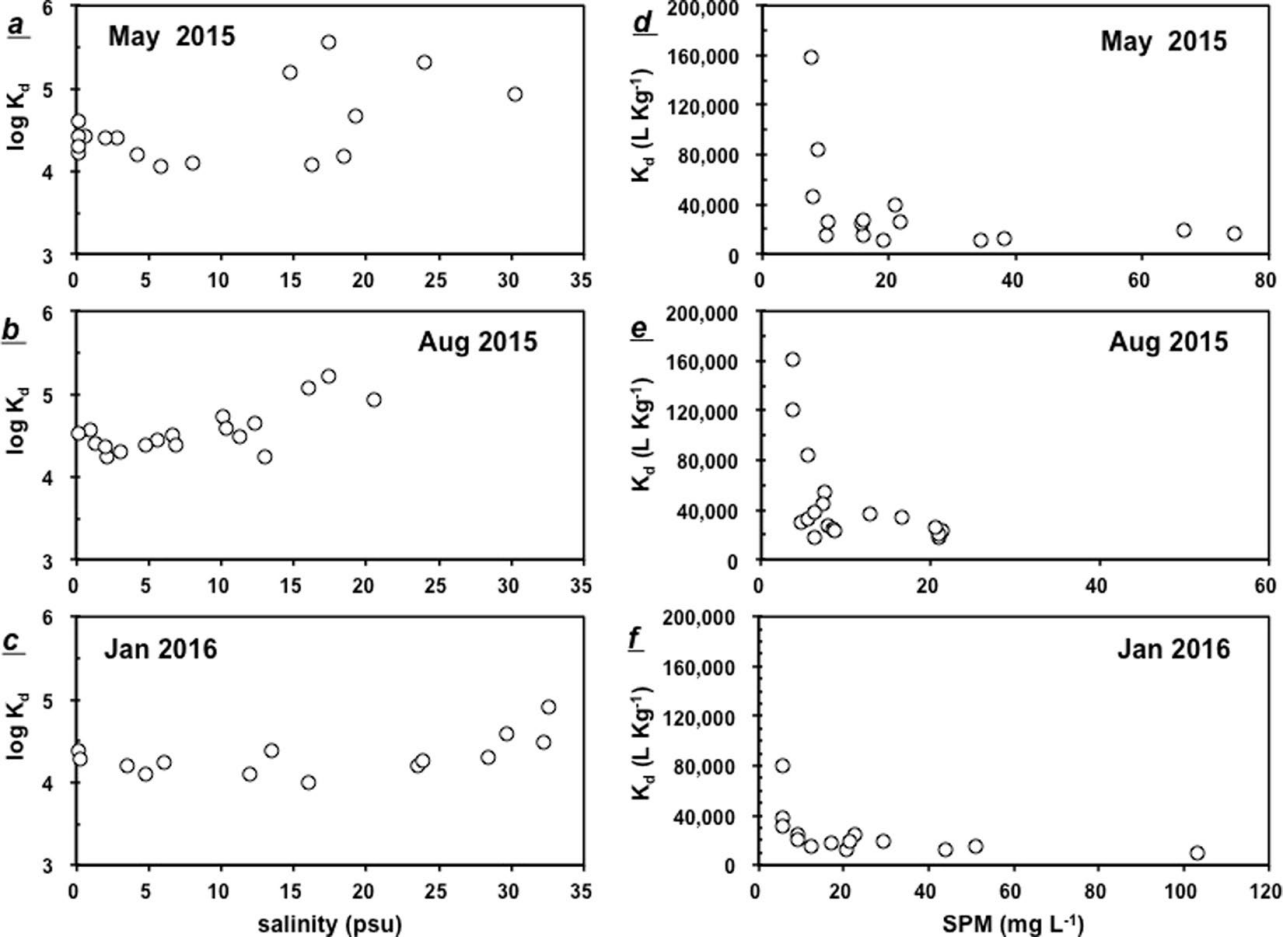
The inorganic distribution coefficients logK_d_ versus salinity (**a**–**c**) and the K_d_ versus the concentrations of SPM in the PRE (**d**–**f**).

The logK_d_ (K_d_, partitioning coefficient) values for inorganic P in the PRE ranged from 3.90 to 5.76 at the surface during the investigation periods throughout the estuary. In addition, the mean logK_d_ values in May (4.50) and August (4.51) were higher than those in January (4.31). The logK_d_ values remained constant along a salinity gradient in the low salinity (0–15 psu) upper estuary, with a slight increase in high-salinity (>15 psu) waters (Fig. 7a–c).

The K_d_ values exhibited an inverse relationship with SPM (Fig. 7d–f) but remained fairly constant when the SPM concentration was higher than 20 mg L^−1^ in May 2015 and January 2016 (Fig. 7d and f).

## Discussion

### Interplay between the Pearl River discharge and estuarine circulation

The physical processes in the PRE demonstrate clear seasonality, with the dominance of freshwater in the wet season and seawater in the dry season due to seasonal exchange between the southwesterly and northeasterly monsoon^25^. The PRE was a typical salt-wedge estuary in the wet season as saline water intruded into the estuary at the bottom and the freshwater outflow dominated at the surface. The freshwater input stratified the water column during the wet season. In comparison, the freshwater discharge declined dramatically, saline water from the SCS dominated in the estuary, and strong vertical mixing occurred in the dry season.

During the study period, the PR discharge (the sum of three tributaries) varied from ~10,000 m^3^ s^−1^ in January 2016 to ~13,700 m^3^ s^−1^ in May 2015 and ~13,400 m^3^ s^−1^ in August 2015. In January 2016, the PR discharge (~10,000 m^3^ s^−1^) was much higher than in a normal year (~2000 m^3^ s^−1^)^28^ due to large storm-induced river runoff. This led to lower salinity in January 2016 (~8 psu) at the HM outlet (Station 4) than in a normal year (~12 psu)^28^, and a strong salinity front occurred in January 2016. Although the PR discharge was higher than normal in January, the salinity distribution in the estuary among the three cruises was still reflective of seasonality. The salinity decreased to ~0.7 psu at Station 4 in May 2015 and ~1.6 psu in August 2015 due to larger river runoff. As shown in the contour plots of temperature, salinity and ∆*t* calculated, the water column was stratified in the estuary during May and August 2015 (Fig. 2).

### Seasonality of nutrients, Chl *a* and suspended materials

Chl *a*, SPM and nutrient concentrations varied significantly during our study periods in the PRE. Chl *a* levels in the estuary increased from January to August and reached a maximum in August 2015 (Fig. 6, Table 1). The temporal variations in Chl *a* had an opposite pattern to those of SPM (Table 1), suggesting that phytoplankton growth was primarily light-limited, since strong vertical mixing induced sediment resuspension and consequently reduced light levels in the water column. Ho *et al*. (2010)^29^ reported that low phytoplankton biomass was primarily attributed to strong vertical mixing in coastal waters, which not only diluted phytoplankton biomass but also led to light limitation. The same results were observed in the tropical Gulf of Carpentaria in winter^30^. Hence, light limitation induced by strong vertical mixing might be responsible for the lower Chl *a* level in the PRE in January 2016.

NO_3_^−^ was the primary component of DIN in January and August, and ammonium was the primary component in May 2015 in the estuary, which was similar to previous observations^28,31^. In our cruise in May 2015, the results of nitrogen isotope analysis suggested that local sewage with high levels of DIN and DIP was a significant source of nutrients in the uppermost estuary (S < 3 psu) in May 2015^32^. The DIN and DSi loading to the estuary was closely related to freshwater discharge, as indicated by the significant relationship between DIN and DSi vs. salinity (Fig. 3).

### Partitioning of P between dissolved and particle phases

The K_d_ approach can be used to quantify the partitioning of P between the particle and dissolved phase and evaluate its particle reactivity^33^. The logK_d_ levels are comparable to the 4.51–4.66 found in the Humber estuary and the 4.62 in the Amazon estuary^34^. A significant correlation between K_d_ with SPM concentrations in the PRE with relatively low levels of SPM (Fig. 7d–f) showed that P was a highly particle-reactive element, and the particle concentrations regulated the particle-dissolved interactions of inorganic phosphorus. Similar results were documented in other estuaries with low SPM levels^35^. In contrast, the dependence of phosphate particle-dissolved interactions on SPM decreases in regions with high SPM concentrations^34^.

Froelich (1988)^36^ suggests that the adsorption of DIP by particles proceeds via a two-step mechanism. The first step, adsorption/desorption on the surface, exhibits fast kinetics (minutes-hours). The second step involves the diffusion of phosphate into the interior of particles and occurs over a much longer time-scale (days to months). Morris (1990)^37^ notes that chemical processes within estuaries are unlikely to reach equilibrium if the kinetics of reactions are slower than the water flushing time. The flushing time of the PRE is less than 3 days^38^, similar to the Tanshui Estuary (approximately 5 to 9 days)^35^, indicating that the phosphate adsorption/desorption processes between dissolved and particulate phases in the PRE might not reach equilibrium, and phosphate should mostly be adsorbed onto the surface of particles that settled out of the water column in the upper estuary with low salinity due to the short flushing time. In the PRE, the SPM levels were closely related to hydrodynamics. In spring and summer, phytoplankton uptake of DIP increased in the low estuary where high Chl *a* concentrations were observed, converting DIP to PP in high-salinity (>15 psu) waters during spring and summer and resulting in high LogK_d_ (Fig. 7a–c).

### Effect of the Pearl River discharge on phosphorus dynamics

Temporal variations in river discharge had a significant impact on both hydrodynamic conditions and the concentrations and composition of nutrients and PP. In January 2016, the thorough vertical mixing resulted in higher concentrations of TPP than in May and August 2015. In spring and summer, stratification not only reduced the resuspension of the SPM at the bottom, but also favored the settlement of the SPM carried by the freshwater. This suggestion was supported by a considerable decrease in the SPM concentration from 67 mg L^−1^ to 10 mg L^−1^ in May and from 21 mg L^−1^ to 9 mg L^−1^ in August 2015. The TPP delivered by the freshwater was settled with SPM in the uppermost estuary (S < 5 psu), as indicated by a sharp decrease (3.29 μM) in the TPP concentration at the surface in the uppermost estuary (S < 5 psu) in May 2015, and a decrease of 1.39 μM in August 2015 (Fig. 3).

As a result, the TPP concentration was low (~0.3 μM) in the water column, especially PIP. The TPP played a limited role in relieving potential P limitation in the PRE even if the remaining TPP could be converted to DIP in May and August. The TPP concentrations (on average 1.09 μM) in the PRE was comparable to some tropical estuaries in Hainan Island in China (0.99 μM)^39^. The TPP levels were lower than in some estuaries that are significantly affected by human activities, such as the Humber estuary (19.1 μM)^34^ and the Yangtze River estuary (3.02 μM)^40^.

In spring and summer, a reduction in the SPM enhanced the light levels in the water column, favoring the phytoplankton growth and leading to high Chl *a* concentrations (Fig. 6). Therefore, the phytoplankton uptake might cause the DIP drawdown. In this study, the two end-member mixing model was used to differentiate the physically induced alterations in DIP from the biological uptake in the study area.

**Figure 8:**
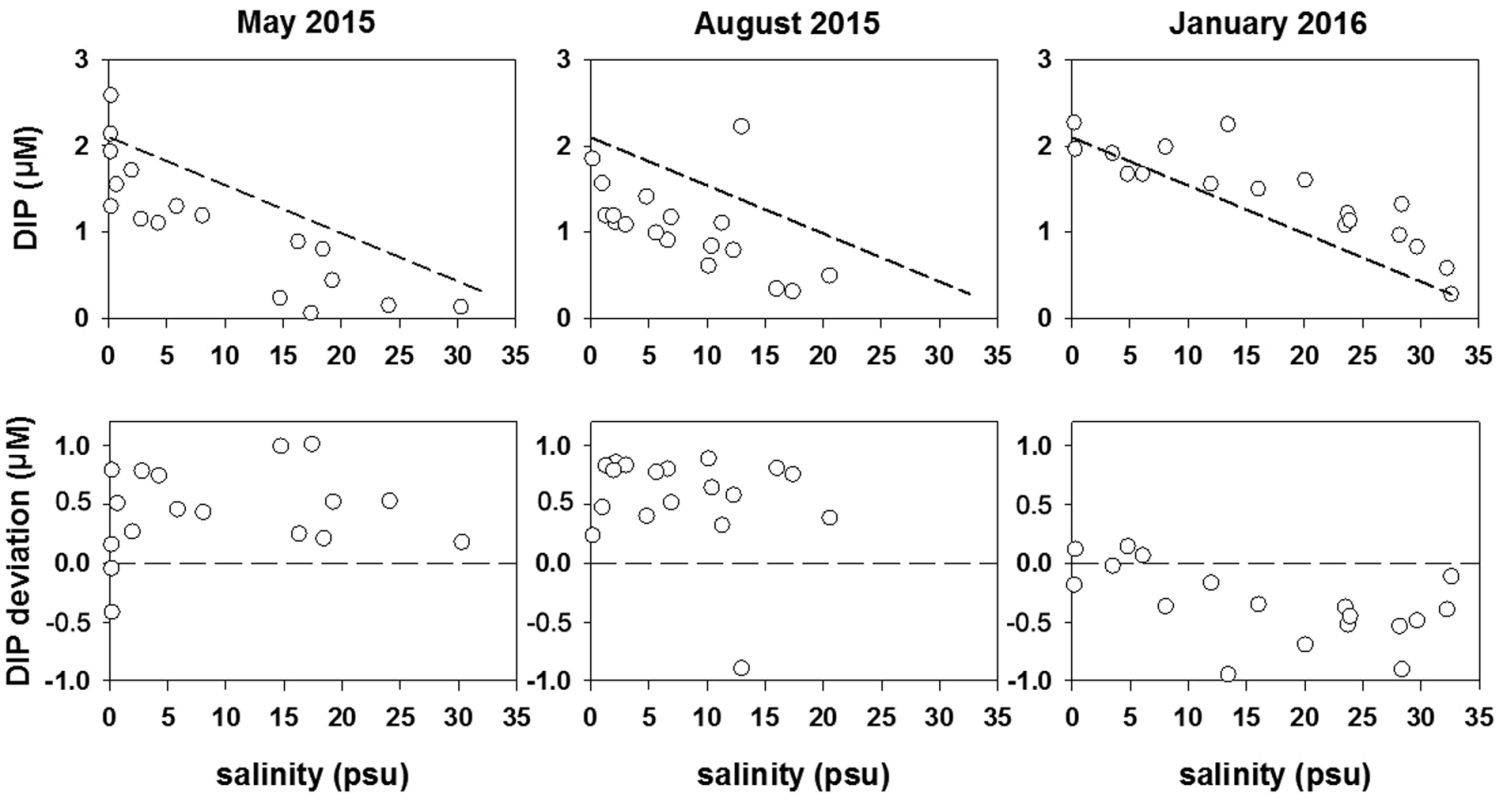
The DIP concentrations versus salinity at the surface in the PRE in May 2015, August 2015 and January 2016. The dashed line represents the theoretical mixing line between freshwater and seawater endmembers in the plot of DIP vs salinity. The DIP deviation denotes the difference between the ambient DIP concentrations and the DIP concentrations predicted by the two end-member mixing model. The dashed line represents no deviation in DIP in the plot of DIP deviation vs salinity. The positive and negative deviations represent the DIP drawdown was caused by biological uptake strong vertical mixing, respectively.

In May and August 2015, positive deviations were observed at most stations, while negative deviations appeared in January 2016 (Fig. 8), suggesting that the DIP drawdown was caused by biological uptake in the spring and summer while the DIP addition that occurred in winter was likely due to the replenishment of DIP from the bottom induced by strong vertical mixing. Based on the estimate by the two end-member mixing model, the biological uptake led to a DIP decline of 0.43 ± 0.37 μM in May and 0.56 ± 0.42 μM in August at the surface. This was responsible for a sharp increase in the relative contribution of DOP to TDP and an extremely high ratio of DIN:DIP in the high salinity waters (S > 15 psu) in May 2015 (Figs 3 and 4). According to the Redfield ratio (106:1 mol mol^−1^) of C:P, the carbon uptake by phytoplankton at the surface was estimated to be 547 μg C L^−1^ in May and 712 μg C L^−1^ in August, respectively. In contrast, the replenishment of DIP from the bottom due to strong vertical mixing resulted in an increase (0.34 ± 0.21 μM) in DIP in winter.

The DOP concentrations exhibited opposite seasonal pattern to the freshwater discharge. In January, there was a clear gradient in the DOP concentration with increasing salinity. In August 2015, when high river discharge occurred, the DOP concentration was very low (~0.15 μM) in the lower estuary. These results implied that the DOP mainly originated from local sewage in the uppermost estuary near the freshwater end-member, rather than the river discharge. The low DOP levels in spring and summer were likely caused by the freshwater dilution and/or the biological uptake. Phytoplankton utilize DOP as a P source to sustain their growth only when inorganic P is deficient^9,10^. However, DIP concentration (~0.40 μM) in the lower estuary was higher than the threshold value (0.1 μM) to limit phytoplankton growth^41^ in August 2015. Hence, the DOP drawdown in summer was more likely due to the freshwater dilution. Consequently, DOP played a limited role in reliving the P limitation of phytoplankton growth as an alternative phosphorus source in the wet season because of the low DOP concentration.

## Conclusion

The Pearl River discharge plays an important role in regulating the dynamics of various P species. All types of phosphorus (DIP: ~1.43 μM, DOP: ~0.49 μM, TPP: ~1.50 μM) were higher in winter due to the replenishment of bottom phosphorus induced by strong vertical mixing and low biological utilization, compared to those in spring and summer. In contrast, the Pearl River discharge input created stratification in the water column in spring and summer, which not only restricted the replenishment of phosphorus in particulate and dissolved forms at the bottom to the surface but also favored the settlement of SPM and increased light levels in the water column. As a result, phytoplankton growth improved, leading to a DIP decline of 0.43 ± 0.37 μM in May and 0.56 ± 0.42 μM in August at the surface. As a result, phosphorus concentrations (DIP: ~1.07 μM, DOP: ~0.18 μM, TPP: < 1.00 μM) were lower in spring and summer than those in winter. Our findings helped us to better understand processes regulating phosphorus dynamics in the PRE.

## Methods

### Sampling and analytical methods

Three cruises were carried out during different seasons in May 2015 (spring), August 2015 (summer) and January 2016 (winter). For each cruise, 18 stations were visited, including the HM Channel (the head of the estuary) in the upstream area to LDY (Fig. 1). Water temperature and salinity were measured using a WTW MultiLine F/Set3 multi-parameter probe. Water samples were collected from the surface and near bottom (~3 m above the bottom layer) waters using a 5-L polymethyl methacrylate water sampler. In the study area, the wet season was from April to September, while the dry season was from October to March.

The water samples for dissolved inorganic nitrogen, silicate, various P species and SPM were immediately filtered through glass fiber filters (GF/F). The filters were used to determine PIP, TPP and SPM. The filtrate was used to measure DIN, DSi and dissolved phosphorus. The filtrates and filters were immediately stored at –20 °C until analysis.

The determination of NO_2_^−^ is based on the reaction of NO_2_^−^ with an aromatic amine, and the product is quantified by spectrophotometry^42^. NO_3_^−^ and NH_4_^+^ were measured by the Cu-Cd column reduction method and the indophenol blue color formation, respectively^42^. The concentration of DIN is the sum of NO_3_^−^ NO_2_^−^ and NH_4_^+^. DIP was measured by the ascorbic acid method^42^. TDP was measured as DIP after digestion of the sample with sodium persulphate in an autoclave (120 °C for 0.5 h)^42^. DOP concentrations were calculated from the difference between TDP and DIP. DSi was analyzed using molybdate, oxalic acid and a reducing reagent^42^. The analytical precision for NO_3_^−^, NH_4_^+^, DIP, DSi and TDP was <5%.

TPP was measured using the methods described by Aspila *et al*. (1976)^43^. The filters were dried at 45 °C before TPP, PIP and SPM were determined. The samples for TPP were combusted at 550 °C for 2 h and then extracted with 1 μM HCl for 16 h. The samples for PIP were extracted with 1 μM HCl for 16 h. TPP and PIP concentrations were obtained by measuring DIP with a spectrophotometer after extraction. The analytical precision of TPP, PIP and POP was <5%^39^. The concentration of POP was estimated by subtracting PIP from TPP. The TP was calculated as the sum of TPP and TDP. SPM was estimated by the weight difference of the GF/F filter before and after filtration.

The Chl *a* was collected on a GF/F filter, extracted in 10 ml of 90% (v/v) acetone in the dark at 4 °C for 14–24 h, and was then measured before and after acidification with 1 μM HCl using a Turner designs Trilogy laboratory fluorometer^44^.

### Two end-member mixing model

The two end-member mixing model was based on mass balance equations for salinity and the fraction of two water masses.

$${{\rm{f}}}_{1}+{{\rm{f}}}_{2}=1$$

$${{\rm{S}}}_{1}{{\rm{f}}}_{1}+{{\rm{S}}}_{2}{{\rm{f}}}_{2}={\rm{S}}$$

where f_1_ and f_2_ were the fractions of the freshwater and seawater, respectively, and S_1_ and S_2_ were the salinity of the two end-members, respectively. Hence, the specific phosphorus concentration (P_m_) predicted by the two end-member model could be calculated as follows:

$${{\rm{P}}}_{{\rm{m}}}={{\rm{P}}}_{1}{{\rm{f}}}_{1}+{{\rm{P}}}_{2}{{\rm{f}}}_{2}$$

where P_1_ and P_2_ were the specific phosphorus concentrations of the two end-members. If we defined

$${\rm{\Delta }}{\rm{P}}={{\rm{P}}}_{{\rm{m}}}-{\rm{N}}$$

where N represented the ambient phosphorus concentration in the water sample. ∆P was the difference between the predicted value and the ambient value; a negative value of ∆P indicated the DIP addition, and a positive value indicated DIP drawdown by biological uptake.

The freshwater end-member was obtained by averaging salinity and phosphorus concentrations at Station 1 over the three cruises. The seawater end-member was based on the salinity and phosphorus concentration at Station 18 in January 2016. The salinity and phosphorus concentration were 0.17 psu and 2.09 μM, respectively, for the freshwater end-member and 32.61 psu and 0.28 μM, respectively for the seawater end-member.

### Partitioning coefficient for P

The distribution coefficient (K_d_) defines the ratio of the adsorbed or particulate concentration to dissolved concentration of a chemical constituent^45^ and is of fundamental significance to understanding the geochemical and contaminant fluxes in estuaries and coastal waters. The interpretation of solid-solution interactions can be quantified using the conditional distribution coefficient, K_d_^34^, given by:

$${{\rm{K}}}_{{\rm{d}}}={{\rm{C}}}_{{\rm{p}}}/{{\rm{C}}}_{{\rm{d}}}/[{\rm{SPM}}]$$

where C_p_ (w/w) is the concentration of PP in a given pool, such as organic, inorganic, or total P pools (all in µM); C_d_ is the concentration of dissolved P in the organic, inorganic, or total P pool (all in µM); and [SPM] is the concentration of SPM. The K_d_ approach has been widely used in previous studies to examine the partitioning or to model phosphorus adsorption-desorption behavior or trace metal removal/adsorption in different environmental settings^17,46,47^.

### Statistical analysis

The p-value from the correlation analysis was derived from functions in SPSS. A Pearson-test analysis was performed to determine significant differences (p < 0.05) between sample sets.
